# Mechanisms of Tumor Growth Inhibition by Depletion of γ-Glutamylcyclotransferase (GGCT): A Novel Molecular Target for Anticancer Therapy

**DOI:** 10.3390/ijms19072054

**Published:** 2018-07-14

**Authors:** Susumu Kageyama, Hiromi Ii, Keiko Taniguchi, Shigehisa Kubota, Tetsuya Yoshida, Takahiro Isono, Tokuhiro Chano, Taku Yoshiya, Kosei Ito, Tatsuhiro Yoshiki, Akihiro Kawauchi, Susumu Nakata

**Affiliations:** 1 Department of Urology, Shiga University of Medical Science, Shiga 520-2192, Japan; kubota@belle.shiga-med.ac.jp (S.K.); yoshida9@belle.shiga-med.ac.jp (T.Y.); yoshiki@mb.kyoto-phu.ac.jp (T.Y.); kawauchi@belle.shiga-med.ac.jp (A.K.); 2 Department of Clinical Oncology, Kyoto Pharmaceutical University, Kyoto 607-8414, Japan; iihiromi@mb.kyoto-phu.ac.jp (H.I.); kd16006@poppy.kyoto-phu.ac.jp (K.T.); 3 Central Research Laboratory, Shiga University of Medical Science, Shiga 520-2192, Japan; isono@belle.shiga-med.ac.jp; 4 Department of Clinical Laboratory Medicine, Shiga University of Medical Science, Shiga 520-2192, Japan; chano@belle.shiga-med.ac.jp; 5 Peptide Institute Inc., Osaka 567-0085, Japan; t.yoshiya@peptide.co.jp; 6 Department of Molecular Bone Biology, Graduate School of Biomedical Sciences, Nagasaki University, Nagasaki 852-8588, Japan; itok@nagasaki-u.ac.jp

**Keywords:** GGCT, C7orf24, cancer, cellular senescence, autophagy

## Abstract

γ-Glutamylcyclotransferase (GGCT), which is one of the major enzymes involved in glutathione metabolism, is upregulated in a wide range of cancers—glioma, breast, lung, esophageal, gastric, colorectal, urinary bladder, prostate, cervical, ovarian cancers and osteosarcoma—and promotes cancer progression; its depletion leads to the suppression of proliferation, invasion, and migration of cancer cells. It has been demonstrated that the suppression or inhibition of GGCT has an antitumor effect in cancer-bearing xenograft mice. Based on these observations, GGCT is now recognized as a promising therapeutic target in various cancers. This review summarizes recent advances on the mechanisms of the antitumor activity of GGCT inhibition.

## 1. Introduction

γ-Glutamylcyclotransferase (GGCT, 188 amino acids, 21 kDa) is an enzyme involved in glutathione metabolism [1], and has recently been described to be overexpressed in a variety of cancers (breast, ovarian, cervical, lung, urinary bladder, colon cancers, osteosarcoma, esophageal squamous cell carcinoma, and glioma) and as a critical player in cancer cell proliferation [2]. Indeed, depletion of GGCT inhibits the aggressive phenotype of various cancers. Although GGCT is thought to be a useful molecular target in cancer therapy, the mechanistic details of GGCT in cancer growth have not yet been fully elucidated. In recent years, several findings have described the inhibitory mechanisms of cancer cell proliferation caused by the depletion of GGCT, thereby increasing the attention to the therapeutic inhibition of GGCT in cancer treatment.

## 2. γ-Glutamylcyclotransferase (GGCT)

**Figure 1:**
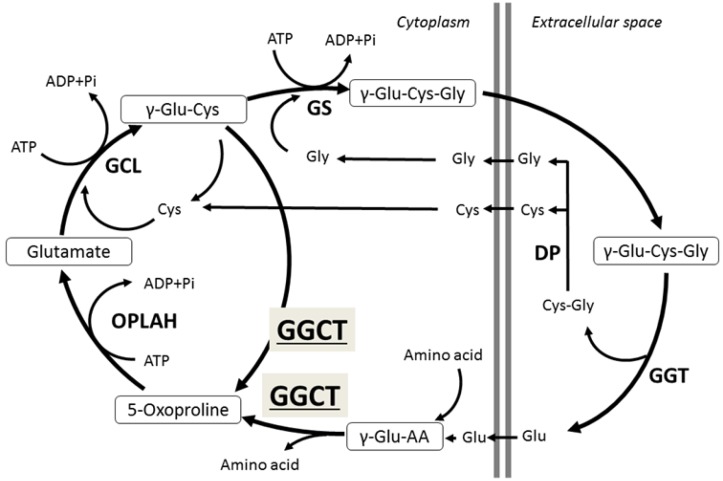
γ-Glutamyl cycle. GGCT, γ-glutamylcyclotransferase; GGT, γ-glutamyltranspeptidase; GCL, glutamate cysteine ligase; GS, glutathione synthase; OPLAH, 5-oxoprolinase; γ-Glu-Cys-Gly, glutathione; DP, dipeptidase.

GGCT is one of the major enzymes in glutathione metabolism [1,3], catalyzing the reaction that leads to 5-oxoproline and free amino acids from the γ-glutamyl peptide. Orthologs of GGCT range from bacteria, plants, and nematodes to higher organisms, and the GGCT gene is highly preserved among a wide range of species. Glutathione is a tripeptide consisting of glutamate, cysteine, and glycine, and present in cells at a relatively high concentration (0.5–10 mM); several roles have been described for this enzyme such as its antioxidant and detoxifying functions. Adequate amounts of the peptides that constitute glutathione (glutamate, cysteine, and glycine) are essential to maintain glutathione at physiological levels [1]. In the 1970s, Meister et al. described the γ-glutamyl cycle that plays a role in the active transport of several amino acids including glutamate, cysteine, and glycine, and GGCT was found to be an essential enzyme in this cycle (Figure 1) [3,4]. Although the GGCT enzyme was isolated more than 40 years ago, its gene locus was unknown until 2008 [5]. Oakley et al. cloned the cDNA encoding human GGCT and found that GGCT was identical to the hypothetical protein chromosome 7 open reading frame 24 (C7orf24), which was previously registered as a putative open reading frame on chromosome 7 (7p15-14).

The comprehensive physiological role of GGCT in normal cells has not been completely elucidated, even though its enzymatic activity has been known since the 1970s. GGCT and γ-glutamyl transpeptidase (GGT) are unique enzymes because both catalyze the γ-glutamyl bond and salvage extracellular glutathione [1]. Imported γ-glutamyl peptides are processed in the cell by GGCT, generating 5-oxoproline and the free amino acids in the γ-glutamyl cycle. The position of GGCT in the γ-glutamyl cycle (Figure 1) suggests that it could play a significant role in regulating the synthesis of glutathione by limiting the availability of γ-glutamylcysteine (γ-Glu-Cys) [5]. GGCT might also have an important cell protective role through an antioxidant effect by glutathione salvage. However, the impact on glutathione levels within the cell have not been reported in previous studies where GGCT levels were manipulated [1]. Further studies are needed to completely clarify the function of GGCT in cancer cells as well as in normal cells.

Several studies have described the distribution of GGCT in normal organs. Oda et al. reported that relatively high levels of GGCT mRNA were expressed in the liver and kidney of rats but were low in other organs [6]. Oakley et al. found higher levels of GGCT in the bladder and salivary glands than in other organs by using GGCT expressed sequence tags (ESTs) in a human EST database [5]. Gromov et al. examined more than 30 normal organ samples and found weak to moderate GGCT protein expression in normal tissues by immunohistochemical analysis using tissue microarrays [7]. Amano et al. also found GGCT protein expression in most normal human tissues, mainly in epithelial cells, by immunohistochemical analysis [8].

Before Oakley’s report, C7orf24 was known as a cancer-related protein. Masuda et al. described C7orf24 as identical to the cytochrome c-releasing factor (CRF21), which is a released substance into the cytoplasm when human leukemia cells U937 are treated with geranylgeraniol, an apoptosis inducer [9]. They presumed that CRF21 could play a critical role in apoptosis signaling because cytochrome c release from mitochondria triggered apoptosis in HeLa cells overexpressing CRF21. Xu et al. identified 46 common cancer signature genes from a pooled DNA array database of previously reported human cancers and reported that one of the highly expressed genes was C7orf24 [10]. Kageyama et al. also described C7orf 24 as an upregulated protein in urothelial cancer specimens by proteomic analysis [11,12]. They conducted proteome differential display with two-dimensional electrophoresis in bladder cancer to search for diagnostic markers or therapeutic targets of urothelial carcinomas [11,12]. They identified 15 highly expressed proteins including C7orf24, and found that C7orf 24 was expressed in 64% and 10% of cancer and non-cancerous tissues, respectively [12].

Accordingly, many studies have recently described high expression levels of GGCT/C7orf24 in various cancers.

## 3. GGCT Upregulation in Cancer

GGCT upregulation in clinical cancer samples has been reported. Gromov et al. performed a large-scale proteome analysis in 123 cases of breast cancer and found that GGCT was highly expressed in neoplastic mammary tissues [7]. They also reported on the association between the expression of GGCT and patient outcome, and showed that patients with high GGCT expression had a poor prognosis. In addition, they studied the expression of GGCT in other cancers and described high expression of GGCT in 58% of uterine cervical cancers, 38% of lung cancers, and 72% of colon cancers. Furthermore, they detected GGCT in the extracellular fluid of mammary glands and suggested the possibility of GGCT as a serum marker for breast cancer. Uejima et al. examined the expression of GGCT mRNA in 40 surgical specimens of osteosarcoma compared with normal human osteoblasts as a control. They reported a high expression (average 8.7 times higher than normal human osteoblasts) in all specimens [13]. Takemura et al. conducted an immunohistochemical examination with the anti-GGCT antibody in 200 specimens of esophageal malignancies [14], and observed increased GGCT expression in 87.5% of esophageal squamous cell carcinomas and 85.0% of high-grade intraepithelial neoplasia; GGCT was still expressed in 17.5% of low-grade intraepithelial neoplasia. Shen et al. reported that GGCT was overexpressed in human glioma specimens [15]. Li et al. also reported that GGCT was upregulated in ovarian cancers and associated with advanced FIGO (International Federation of Gynecology and Obstetrics) stage, lymph node metastases, and ascitic fluid volume in high-grade serous ovarian cancers (HGSCs) [16]. They also observed that GGCT upregulation was related to poor survival in HGSCs. Their multivariate analysis showed that FIGO stage, lymph node metastasis and GGCT expression were independent prognostic factors for overall and progression free survival. Taken together, the data of these studies using clinical samples show that GGCT upregulation is a common event in a wide range of malignant tumors.

The upregulation of GGCT has been reported in a wide range of cancer cell lines including breast, ovarian, cervical, lung, urinary bladder, prostate, colon cancers, osteosarcoma, and glioma cells. However, the detailed role of GGCT in cancer cells is still unclear. To elucidate this, gene transfection and knockdown experiments were performed in previous studies. Kageyama et al. observed increased proliferation when the GGCT gene was expressed in NIH3T3 cells, but did not observe any change in the soft agar assay and focus-forming assay [12]. Azumi et al. reported similar findings in HBL-100 cells, a breast cancer cell line with low GGCT expression [17]. These results strongly suggest that GGCT might not be directly involved in malignant transformation, but in other events that lead to the malignant phenotype.

## 4. Molecular Regulation Mechanism of GGCT Expression in Cancer Cells

Two reports have described the mechanism of regulation of the GGCT gene in normal and malignant cells [18,19]. The first, by Ohno et al., showed that the region located at −371 to +14 bp of the 5′ end of GGCT is important for the activation of its transcription in both cancer (HeLa, MCF7) and non-cancer cells (IMR-90). Sequencing analysis showed that this region has three CCAAT boxes near the transcription start site and a GC box upstream of these CCAAT boxes. NF-Y and Sp1 bind to the CCAAT boxes and to the GC box, respectively, to positively regulate transcription of the GGCT gene. Since promoters with several NF-Y-binding CCAAT boxes were found in genes related to the cell cycle, Ohno et al. suggested that GGCT may also play a role in the cell cycle [18], as confirmed by other studies [19,20,21,22].

In addition, Ohno et al. reported a different structure of the GGCT promoter between normal (ARPE-19, IMR-90) and cancer cells (HeLa, MCF7) [23]. The GGCT gene has a stable heterochromatin structure in normal cells and a euchromatin structure in cancer cells. Therefore, the high expression of GGCT in cancer cells is caused by a structural change in the chromatin of the GGCT gene associated with the oncogenic transformation of the cells [23]. These studies have nicely elucidated the different levels of expression of GGCT in normal and cancer cells.

GGCT might also be regulated by a feedback mechanism due to the metabolites of the γ-glutamyl pathway and/or intracellular oxidative stress and may be controlled by products of the γ-glutamyl cycle such as glutathione [24]. Further studies will clarify the detailed mechanisms of the regulation of GGCT expression.

## 5. Inhibition of Cancer Cell Proliferation and Induction of Cell Death by GGCT Knockdown

In 2007, Kageyama et al. showed that GGCT knockdown inhibited the proliferation of several types of cancer cells, but not of normal human fibroblasts [12]. Similarly, other groups have reported that GGCT depletion inhibited the growth of cancer cell lines in vitro including osteosarcoma [13], lung cancer [19], glioma [15], gastric cancer [20], colorectal cancer [21], and ovarian cancer cells [16]. Two in vivo experiments using xenograft mice have also been performed [25,26]. Hama et al. produced tumor-bearing mice by subcutaneous implantation of EBC-1 cells, a lung squamous cell carcinoma cell line; after local administration of GGCT siRNA to the tumor, using a needle-free jet injection, they observed significant regression of the tumor [25]. Jet injection can be used to inject a drug through the skin by high air pressure, allowing a wide distribution of naked nucleic acid within the injection site. Ran et al. succeeded in systemic cancer treatment targeting GGCT in tumor-inoculated mice via a unique drug delivery system of intravenous administration of siRNA [26]. They established the PEGylated hyaluronic acid-modified liposomal delivery system, and described significant antitumor effects in mice inoculated with adriamycin-resistant MCF7 cells, a breast cancer cell line. Importantly, they also reported that the systemic administration of GGCT siRNA did not affect normal organs such as kidney, heart, lung, liver, and spleen.

Several analyses were performed by different research groups to clarify the role of GGCT in the cell cycle. Lin et al. observed G0/G1 arrest after GGCT knockdown in A549 lung cancer cells [19] with the decrease of G0/G1-associated markers CDK4, CDK6, and cyclin D1. Dong et al. also reported that GGCT knockdown blocked cell cycle progression at the G0/G1 phase in both HCT116 and in SW1116 colorectal cancer cells [21]. Matsumura et al. observed G0/G1 arrest in MCF7 breast cancer cells [22]. In contrast, Zhang et al. analyzed cell cycle change using a gastric cancer cell line, MGC80-3, and reported a significant reduction of cell cycle progression in shGGCT cells, with a reduction of the cell population in the G0/G1 phase and a corresponding increase in the G2/M phase when compared with the control cells [20]. These findings indicate that lack of GGCT suppresses cell cycle progression in different cell lines, but the cell cycle phases in which GGCT depleted cells accumulate may vary according to the cellular contexts, probably due to each mutation spectrum of the cells.

Cell death mechanisms caused by GGCT knockdown are also controversial. Hama et al. observed caspase activity and Annexin V positivity in isolated tumor tissues from tumor bearing mice. They reported that the transduction of siGGCT resulted in higher caspase 3/7 activity and increased Annexin V-positive cells when compared with the transduction of nonspecific siRNAs, suggesting that apoptotic induction may be responsible for cell death in GGCT silenced cells [25]. Lin et al. reported that cleaved PARP increased in shGGCT cells, suggesting that silencing GGCT also induced cell apoptosis through the cleavage of PARP [19]. Other groups have also described apoptotic cell death by GGCT knockdown [20,21,26]. In contrast, Li et al. reported no change in cell apoptosis after interference of GGCT expression [16]. Matsumura et al. showed that apoptotic signaling molecules such as caspases and PARP were not activated by GGCT knockdown in MCF7 cells [22]. Therefore, further studies are needed to clarify the precise cell death mechanism by GGCT depletion by considering the mutation background of specific cell types.

Only one article has been reported miRNA targeting the *GGCT* gene [27]. Zhang et al. found decreased expression levels of miR-194 in gastric cancer tissues when compared with the adjacent non-cancer tissues, and decreased miR-194 expression in gastric cancer cell lines. Using miRNA target prediction, they found *GGCT* as a target gene of miR-194 and showed that overexpression of miR-194 inhibited proliferation of MGC-803 gastric cancer cells.

## 6. Intracellular Changes Caused by GGCT Depletion

### 6.1. Morphological Changes

One study described the role of GGCT in cell morphology, motility, and invasion. Uejima et al. reported that downregulation of GGCT expression induced clustering of human osteosarcoma (HOS) cell lines [13]. Parental and control (control siRNA) HOS retained their original spindle shape and continuously proliferated with few cell–cell contacts until confluent. In contrast, siGGCT–HOS cells became polygonal to cuboidal in shape and tended to cluster via cell–cell contacts. Furthermore, DNA microarray analysis clarified that cell adhesion-related molecules such as integrins and cadherins were increased. In the same study, silencing GGCT reduced cell invasions as well as cell motility, thus suggesting the potential involvement of GGCT not only in tumor growth, but also in invasion and/or metastasis [13].

### 6.2. Cellular Senescence

In addition to the functions already described, GGCT has also been involved in cellular senescence. Matsumura et al. noticed that GGCT-depleted cells exhibited a pronounced flat and enlarged morphology, a characteristic phenotypic change associated with cellular senescence. They found that GGCT knockdown significantly induced cellular senescence with SA-β-gal staining, a specific marker for senescent cells, in various cancer cells including MCF7, MDA-MB-231 (breast cancer), PC3, LNCaP (prostate cancer), HeLa (cervical cancer), and A172 (glioblastoma) [22]. They found that p21^WAF1/CIP1^ and/or p16^INK4A^ were upregulated in all cell lines tested. Moreover, simultaneous knockdown of p21^WAF1/CIP1^ restored cell cycle arrest, attenuated cellular senescence, and rescued the subsequent growth inhibition in GGCT silenced MCF7 cells [22]. In addition, blockade of senescence by p21^WAF1/CIP1^ or p16^INK4A^ knockdown led to a significant reduction of non-apoptotic cell deaths, suggesting that cell death upon GGCT depletion was, at least in part, mediated by cellular senescence following the sustained cell cycle arrest. These findings demonstrated that induction of cellular senescence mediated by the upregulation of CDK inhibitors was a major event underlying the antiproliferative effect caused by GGCT depletion in cancer cells. Similarly, upregulation of CDK inhibitors was also reported by Dong et al. [21], who observed that GGCT depletion caused an increase of p21 and p27, and a decrease of cyclin E in SW1116 colon cancer cells.

### 6.3. Autophagy

**Figure 2:**
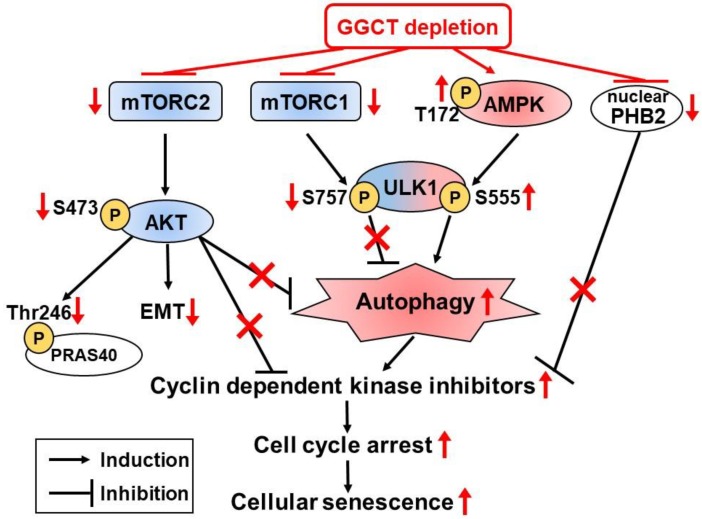
Hypothetical mechanisms of tumor growth inhibition by depletion of GGCT. Promoting effects are illustrated with black arrows, inhibiting effects are illustrated with black T, and the influences by GGCT knockdown are indicated in red.

Recently, Taniguchi et al. published an attractive work concerning autophagy induction via GGCT knockdown [28]. They investigated the levels of LC3-II, an autophagosome related protein, in MCF7 and PC3 cells transduced with GGCT targeting siRNA or non-target siRNA. The study revealed that GGCT knockdown increased the levels of LC3-II and autophagosome formation, which could be attenuated by simultaneous knockdown of Atg5, a critical mediator of autophagy induction. Conversely, overexpression of GGCT in NIH3T3 cells under serum starvation, a model for autophagy induction, significantly blocked LC3-II induction and recovered proliferation, highlighting the role of GGCT in the enhancement of cell growth even in such a severe cellular circumstance. They also showed that blocking autophagy (1) attenuated GGCT depletion-mediated CDKI upregulation and cell cycle arrest; (2) rescued cell growth inhibition caused by GGCT knockdown; and (3) inhibited cellular senescence induced by GGCT knockdown; in addition, they found that GGCT depletion activated the AMPK-ULK1 pathway and led to the inactivation of the mTORC2-AKT pathway in cancer cells. These results indicate that autophagy plays an important role in growth inhibition induced by GGCT depletion in cancer cells, and also imply the involvement of autophagic cell death in GGCT depletion mediated cell death. As discussed, different hypothetical mechanisms of antitumor activity via depletion of GGCT have been proposed, all summarized in Figure 2.

### 6.4. Changes of Intracellular Signaling Pathways

Several groups have recently reported alterations of intracellular signaling pathways after GGCT knockdown. Shen et al. found that GGCT knockdown resulted in a loss of Notch1 and Notch2 expression in T98G and U251 glioma cell lines [15]. When Notch was inhibited in T98G cells, GGCT overexpression could not rescue the suppressions of cell proliferation and colony formation. In addition, the study showed that phosphorylated AKT was correlated with GGCT expression levels. These findings suggest that Notch signaling together with AKT contribute to the effects of upregulated GGCT in human glioma cells. Dong et al. studied the changes of intracellular signaling pathways relating to apoptosis using an antibody array [21]. Their result indicated that GGCT silencing downregulated the phosphorylation of PRAS40 (proline-rich AKT substrate of 40 kDa) at Thr246 (Figure 2) and upregulated the cleavage of PARP at Asp214, and that cancer growth control by GGCT occurred, at least in part, through the regulation of apoptosis-related proteins.

### 6.5. Epithelial Mesenchymal Transition (EMT)

A recent research was recently published on the involvement of GGCT in EMT [16]. Li et al. reported that GGCT was highly upregulated in ovarian cancers, and high expression of GGCT was associated with poor survival. Moreover, they reported that GGCT knockdown suppressed proliferation, colony formation, migration, and invasion of ovarian cancer cells in vitro, and GGCT silencing inhibited tumor growth and spreading in vivo. Based on these findings, they investigated the expression of EMT markers; the expression levels of E cadherin were markedly upregulated whereas those of vimentin and Twist were significantly decreased in GGCT depleted cells. They also reported that the PI3K/AKT/mTOR signaling pathway was suppressed in GGCT silenced cells and enhanced in GGCT overexpressing cells. After treatment with the PI3K signaling pathway inhibitor LY294002, the EMT caused by GGCT overexpression was suppressed, thus highlighting the contribution of GGCT to the EMT via the PI3K/AKT/mTOR signaling pathway (Figure 2).

### 6.6. GGCT Interacting Proteins

Proteins binding to GGCT have been discovered only recently. To identify proteins interacting with GGCT, Taniguchi et al. performed yeast two-hybrid screening and found that the PHB2 protein bound to GGCT, a result also confirmed by the immunoprecipitation of MCF7 cell lysates using an anti-GGCT monoclonal antibody [29]. They also showed that nuclear expression of PHB2 in MCF7 cells was decreased after GGCT knockdown, and that overexpression of PHB2 inhibited p21 upregulation, caused by GGCT knockdown. A chromatin immunoprecipitation assay revealed that nuclear PHB2 proteins bound to the p21 promoter, and that this interaction was abrogated by GGCT knockdown. Moreover, knockdown of PHB2 alone led to significant upregulation of p21 and mimicked the subsequent cellular events induced by GGCT depletion including G0/G1 arrest, cellular senescence, and growth inhibition in a p21-dependent manner (Figure 2). Although further investigations are needed to clarify the underlying molecular mechanisms between PHB2 subcellular localization and GGCT interaction, these findings suggest the existence of a new function for GGCT, distinct from a simple enzymatic role in glutathione metabolism.

## 7. GGCT Fluorogenic Probes, LISA-4 and LISA-101

Recently, Yoshiya et al. developed novel GGCT fluorogenic probes, LISA-4 and LISA-101, to visualize GGCT activity in cells [30,31]. The singular substrate preference of this enzyme has long hampered its chemical probe development. Since γ-glutamyl amino acids (γ-Glu-Xaa) are known as specific substrates for GGCT, they assumed that γ-Glu-Xaa could become a GGCT fluorogenic probe if Xaa contains a latent fluorophore and releases an intact fluorophore upon enzymatic cleavage. Based on this assumption, they designed a dipeptide compound γ-Glu-Ser(CO-4-methylumbelliferone) (LISA-4). When an α-amino group of Ser is liberated by GGCT, the Ser derivative releases the intact fluorophore, methylumbelliferone (MU). As a result, fluorescence was regained from LISA-4 when the probe was incubated with GGCT, thus successfully developing a first-generation fluorogenic probe for GGCT [30].

Although LISA-4 enabled the visualization of GGCT activity, the carbonate bond in this molecule was not sufficiently stable under neutral conditions and hampered its application to cell-based assays. Yoshiya et al. improved LISA-4 and developed a new fluorescent probe, LISA-101 [31], by modifying the structure. They designed a stable GGCT probe by modifying the structure of LISA-4. The connection of the fluorophore with the side chain of Xaa was replaced by an *N*-alkyl urethane structure, more stable than the carbonate bond used in LISA-4. To use the probe in cell-based imaging, the fluorophore was changed to resorufin (emission λmax: 590 nm), which has a longer fluorescence wavelength than the MU (emission λmax: 445 nm) used in LISA-4 [31].

Yoshiya et al. proved the applicability of LISA-101 using NHDF (normal human dermal fibroblast) cells, which express low levels of GGCT, and MCF7 breast cancer cells, which express high levels of GGCT [31]. They found that MCF7 cells showed 100-times higher GGCT activity than NHDF cells, thus indicating that LISA-101 is a specific probe for GGCT. Thanks to the research on GGCT probes, LISA-101 is now an established fluorogenic probe that can be used in the compound screening of GGCT inhibitors and will therefore lead to the detection of new promising inhibitors.

## 8. Development of GGCT Inhibitors

Recently, the first cell membrane permeable GGCT inhibitor has been developed by Ii et al. [32]. As the structure of an inhibitor of enzymatic activity often resembles the structure of the natural substrate of the enzyme, 18 compounds structurally similar to γ-glutamyl-l-amino acid were selected as candidates for GGCT inhibition. *N*-glutaryl-l-alanine (GA) was found to be the most potent inhibitor for GGCT by compound screening using the LISA-101 assay, a novel GGCT fluorogenic probe [33,34]. GA was an effective GGCT inhibitor in cell homogenates, but was unable to penetrate through the cell membranes. In order to improve the cell membrane permeability of GA, diester types of GA were made as prodrugs that could efficiently penetrate the cell membrane [33]. Among the candidate compounds, methyl acetoxymethyl GA was selected as the compound with the highest permeability. Methyl and acetoxymethyl bonds were hydrolyzed by intracellular esterase. The newly developed cell permeable GGCT inhibitor was named “pro-GA”. The antiproliferative activity of pro-GA was demonstrated in human cancer cells including MCF7, HL-60 (leukemia), and PC3 cells. In contrast, normal cells were not significantly affected by pro-GA treatment. Moreover, pro-GA administration exhibited significant anticancer effects in a mouse model of PC3 prostate cancer xenograft. No obvious toxicity or change in body weight was observed in the mice treated with pro-GA.

Pro-GA, as a cell membrane permeable GGCT inhibitor, might become a promising novel therapeutic agent against various cancers expressing high levels of GGCT such as breast, ovarian, cervical, lung, urinary bladder, colon cancers, osteosarcoma, esophageal squamous cell carcinoma, and glioma.

## 9. Conclusions

GGCT has attracted the attention of cancer research due to its involvement in the malignant phenotype. The fundamental research on the inhibition of proliferation triggered by GGCT depletion in cancer cells has rapidly advanced over the past few years, leading to new discoveries. Several groups have described GGCT as a key player in cancer proliferation, senescence, apoptosis, morphology, motility and invasion, and autophagy. In addition, pro-GA has been developed as the first cell membrane permeable GGCT inhibitor, thus giving rise to new research for the discovery of anticancer drugs. Although the antitumor effect followed by GGCT inhibition is evident, the assessment of adverse events due to the inhibition of GGCT in normal tissues should be further investigated. Careful investigation is needed to clarify whether changes in intracellular metabolites by GGCT inhibition may cause critical damage in healthy cells. In addition, the specificity of GGCT inhibitors need to be carefully tested. Following appropriate research, molecular targeting of GGCT through small inhibitors might become a promising clinical treatment used in cancer therapy in the near future.

## Abbreviations

Atg5	Autophagy related 5
C7orf24	Chromosome 7 open reading frame 24
CDK	Cyclin dependent kinase
EMT	Epithelial mesenchymal transition
GA	*N*-glutaryl-l-alanine
GGCT	γ-glutamylcyclotransferase
LC3-II	Microtubule-associated protein light chain 3-II
PHB2	Prohibitin 2
Pro-GA	Prodrug of *N*-glutaryl-l-alanine
SA-β-gal	Senescence associated β-galactosidase
